# Predictors of students’ self-reported adoption of a smartphone application for medical education in general practice

**DOI:** 10.1186/s12909-015-0377-3

**Published:** 2015-05-21

**Authors:** Maximilian Sandholzer, Tobias Deutsch, Thomas Frese, Alfred Winter

**Affiliations:** 1 Institute for Medical Informatics, Statistics and Epidemiology, Medical Faculty, University of Leipzig, Härtelstraße 16-18, 04107 Leipzig, Germany; 2 Department of Primary Care, Medical Faculty, University of Leipzig, Philipp-Rosenthal-Str. 55, 04103 Leipzig, Germany

**Keywords:** Smartphone application, ELearning, EHealth, General practice, Independent adoption predictors

## Abstract

**Background:**

Smartphones and related applications are increasingly gaining relevance in the healthcare domain. We previously assessed the demands and preferences of medical students towards an application accompanying them during a course on general practice. The current study aims to elucidate the factors associated with adoption of such a technology. Therefore we provided students with a prototype of an application specifically related to their studies in general practice.

**Methods:**

A total estimation among students participating in a general practice examination at the Leipzig Medical School was conducted in May 2014. Students were asked to answer a structured self-designed questionnaire. Univariable comparisons were made to identify significant differences between those students who reported to have used the application frequently and those who did not. Multivariable binary logistic regression was used to reveal independent predictors of frequent application usage.

**Results:**

The response rate was 99.3 % (n = 305/307). The majority (59 %, n = 180/305) were female students. The mean age was 24.5 years and 79.9 % (n = 243/304) owned a smartphone or tablet computer. Regarding the usage of the provided application, 2.3 % (n = 7/303) did not use the app while 68.0 % (n = 206/303) replied to have used it more than five times. Frequent users significantly differed from non-frequent users with regard to being female rather than male, higher mobile device ownership, more frequent exchange about obtaining the course certificate, higher personal interest in new technologies, larger enjoyment of the technology, lower intention to not use smartphone applications in the future, better opinion towards smartphone applications for the profession of a doctor, higher perceived importance of medical applications on the job, higher compatibility of smartphone applications with personal work style, higher perceived relevance of university support and personal benefit of use. Multivariable analysis revealed a set of four variables independently predicting frequent usage: being female, a higher perceived benefit of the supplied application, a higher personal interest in new technologies, and a higher perceived impact of previous experiences on smartphone adoption (Pseudo-R^2^_Nagelkerke_ = 0.245).

**Conclusions:**

Understanding medical students’ adoption of smartphone applications used for educational purposes may provide useful information to guide the implementation process as well as the design of respective applications.

**Electronic supplementary material:**

The online version of this article (doi:10.1186/s12909-015-0377-3) contains supplementary material, which is available to authorized users.

## Background

The smartphone has gained increasing importance in our everyday life. The commercial success of smartphones followed the introduction of the iPhone and android devices in 2007 and 2008 [1, 2]. Due to their functionality and potential, smartphones are gaining importance in healthcare and attract the attention of researchers and developers of healthcare related apps. Recent research has majorly focused on reviewing, testing and developing smartphone apps for topics such as physical activity tracking, patient monitoring, diagnosis and measurement [3–8].

Additionally, the potential of smartphone apps for medical education is being explored [9]. Mobile devices enable learning in context through access to key facts and allow making use of otherwise wasted time [10]. A recent study concluded that while only few e-learning apps exist, they could provide an ideal platform as most of the users are wearing a smartphone on them at all time [11]. This mobility of learning enabled by smartphones explains why students evaluated an app containing medical guideline as a modern way to familiarize oneself with the content, as waiting periods could be effectively utilized [12]. The possibility to immediately access information aids the consolidation of knowledge through repetition and makes mobile devices a valuable addition to traditional learning resources [10]. This is supported by 71.4 % of recently interviewed psychiatry students mentioning that a related app would be a good addition to a textbook [13]. Thus, medical teachers should integrate this supplement into the traditional set of learning tools, and thereby influence students’ perception of that technology’s relevance to transform them into digital academics [14]. In fact, first initiatives were launched by educators [15–17] and significant effects on students’ examination performances were reported [16].

As user acceptance of new technologies is a determinant factor of its success, it is crucial for developers and designers to identify factors that influence adoption of their technology [18]. Given that medical apps have been attracting the interest of researchers on a large scale, surprisingly little research has been conducted to investigate which kind of users in the medical domain adopt this new technology. Outside the healthcare domain several studies have examined the adoption factors of mobile technologies. A study from 2010 has assessed the factors driving the adoption intention of m-learning employing the Technology Acceptance Model [19]. A earlier study researched the indicators of the intention to use a smartphone [20]. In the field of medicine, a study among U.S. physicians investigated the innovation factors that affect a physician’s decision to adopt a smartphone [21].

We previously assessed the demands of students towards and the potential of an app on general practice [9]. A prototype of an general practice app has been developed as a mobile website for medical education in Germany. This app has been provided to students attending a lecture series on general practice as a tool to acquire or intensify their knowledge on the go and conduct mock up tests to prepare for the examination. Based on the gap in literature on understanding the factors influencing the adoption of such a mobile technology for medical education, we aimed to investigate (1) which characteristics distinguish those students who adopted the technology from those who did not and (2) which factors are relevant independent predictors of adoption in the sense of frequent use.

## Methods

### Sampling and design

The present cross-sectional study was conducted at the Leipzig Medical School in May 2014. At the beginning of the lecture series in general practice (8^th^ semester of 12) the web-based prototype of the new smartphone application and its functionality was introduced to the participating students, who were encouraged to use it as an additional learning tool. At the end of the lecture series, immediately prior to the respective mandatory multiple-choice examination, a structured anonymized questionnaire was distributed by person. Before handing out the questionnaires, all students were notified about the purpose of the voluntary survey. Regarding the regulations of the ethical board of the Leipzig Medical School, no ethical approval was necessary.

### Questionnaire

The questionnaire was developed by an interdisciplinary team consisting of one economist, a psychologist, a general practitioner and a computer scientist and has been reviewed by several general practitioners. Questionnaire content was motivated by the Diffusion of Innovations Theory as developed by Rogers and the adjusted Technology Acceptance Model as presented by Kim in 2008 [22, 23]. Prior to the survey, we piloted the questionnaire with a selection of students to verify the general understandability leading to further minor revisions. The final version collected data in the following areas: general information (gender, age, etc.), devices owned, possible determinants of medical app usage, usage behavior of the application provided, lecture attendance, usage of medical textbooks and frequency of exchange with other students on passing the exam. The section that specifically aimed at surveying possible predictors aside of medical app usage included questions on students’ general attitude towards new technology and smartphones, their future intention of using smartphone applications, job relevance, perception of other students behavior, previous experience and relevance of university promotion, outside trends and student involvement in development of smartphone applications. An English translation of the questionnaire items relevant for the present analysis is given in Appendix 1 (Additional file 1).

### Short description of the provided smartphone application

The provided smartphone application was developed on the basis of a general practice textbook [24] considering the results of an earlier study on student demands towards an application in the field of general practice [9]. The web-based prototype provided specialist texts, information on medicaments, differential diagnostic algorithms and lecture notes. In addition to digitalized textbook content, the prototype also contained selected scientific articles available for download and a multiple-choice mock examination as well as links between relational content. A screenshot of the user interface is presented in Appendix 2 (Additional file 2).

### Statistical analysis

The data was analyzed with IBM SPSS Statistics 22 for Windows. Frequencies were presented as %_valid_ (n_absolute_/n_valid_), as the number of valid values differed from item to item. Continuous variables were presented as mean ± standard deviation (SD). Frequencies were compared using the Chi-square test. Continuous variables were compared using the Mann–Whitney *U* test. Multivariable binary logistic regression was used to reveal predictors of a more frequent use of the provided smartphone application. Therefore a dichotomous outcome variable was created differentiating those students who used the app five times or less from those who used it more than five times. All relevant variables identified by univariable analyses (Chi-square test for categorical, univariable logistic regression for metric variables) as being potentially associated with the outcome variable on a significance level of p < 0.20 as a screening criterion were entered into stepwise forward (LR) logistic regression. Statistical significance was assumed for p < 0.05.

## Results

### Sample characteristics and device ownership

We received 305 out of 307 completed questionnaires (response rate 99.3 %). 59.0 % (n = 180/305) of the participants were female. Mean age was 24.5 ± 2.7 years (range 21 to 35, n = 297). We found that 75.3 % (n = 229/304) owned a smartphone, 39.8 % (n = 121/304) a tablet computer, 91.1 % (n = 277/304) a laptop and 17.1 % (n = 52/304) a desktop computer. Furthermore, 77.0 % (n = 234/304) of the surveyed students reported to have private internet access and 79.9 % (n = 243/304) of the students owned a mobile device (smartphone and/or tablet computer). We found no differences in mobile device ownership between males and females (82.4 % (n = 103/125) vs. 78.2 % (n = 140/179); p = 0.370).

### Usage frequency of the provided smartphone application

A majority of the students used the app frequently in the period before the exam: 2.3 % (n = 7/303) did not use the app at all, 1.7 % (n = 5/303) used it once, 28.1 % (n = 85/303) used it between two to five times, 33.3 % (n = 101/303) used it between six and ten times and 34.7 % (n = 105/303) used it more than ten times.

### Univariable analysis

To analyze factors associated with a more frequent use of the app, we compared the students who stated to have used the app five times or less (32.0 %, n = 97/303) with those that used it more than five times (68.0 %, n = 206/303) regarding a total of 21 potentially relevant variables.

In Table 1 the group comparisons regarding the surveyed student general information and their orientation towards performance in the lecture are presented. Table 2 reports the comparisons of the agreement for the set of attitudinal variables potentially influencing adoption. The group comparisons concerning the students’ perceptions of the benefit of the provided application and its ease of use are shown in Table 3.

**Table 1:** Student general properties (1 to 3) and performance orientation (4 to 6) – group comparisons based on usage

Variable	Valid N	Five times or less used	More than five times used	p
		N_a/_N_v_ (%)^a^	N_a/_N_v_ (%)^a^	
1. Gender: Female	303	46/97 (47.4)	132/206 (64.1)	Chi^2^ = 7.548, **p = 0.006**
2. Age in years (mean ± SD; median)	296	24.7 ± 2.7; 24	24.4 ± 2.6; 24	p = 0.360*
3. Mobile device ownership: Yes	303	70/97 (72.2)	172/206 (83.5)	Chi^2^ = 5.265, **p = 0.022**
4. Lecture attendance: More than five times	295	31/96 (32.3)	87/199 (43.7)	Chi^2^ = 3.523, p = 0.061
5. Medical textbook usage: More than five times	295	30/96 (31.3)	75/199 (37.7)	Chi^2^ = 1.171, p = 0.279
6. Exchanged about certificate: More than five times	293	17/95 (17.9)	65/198 (32.8)	Chi^2^ = 7.104, **p = 0.008**

^a^N_aboslute_/N_valid_ (percent), unless otherwise indicated

*Mann Whitney *U*-Test

**Table 2:** Students’ agreement with attitudinal variables potentially influencing the adoption – group comparisons based on usage

Variable	Valid N	Five times or less used	More than five times used	p
(*Likert scale from 1 = “strongly disagree” to 5 = “strongly agree“*)		Mean ± SD	Mean ± SD	(*U*-Test)
7. I always belong to the first ones that use a new technology.	301	2.3 ± 1.0	2.8 ± 1.1	**< 0.001**
8. I do not enjoy the usage of smartphones and smartphone applications.	303	2.5 ± 1.3	2.1 ± 1.0	**0.004**
9. I will definitely use medical smartphone applications less in the future.	299	2.2 ± 1.0	1.9 ± 0.9	**0.006**
10. Using medical smartphone applications for the profession of a doctor is good.	301	3.8 ± 1.0	4.2 ± 0.8	**< 0.001**
11. Medical smartphone applications are used frequently among my fellow students.	299	3.3 ± 1.1	3.5 ± 0.9	0.164
12. I consider myself unconfident when handling smartphones and smartphone applications.	298	2.1 ± 1.0	2.0 ± 1.0	0.177
13. Having medical smartphone applications is important in my working environment.	301	2.5 ± 1.0	2.8 ± 1.0	**0.005**
14. Using medical smartphone applications does not fit personal way of working.	301	2.9 ± 1.1	2.4 ± 1.1	**0.001**
15. I believe that the usage of new media does not depend on the attitude of an individual.	299	2.2 ± 0.9	2.3 ± 0.9	0.095
16. Whether or not one uses a smartphone application essentially depends on previous related experiences.	297	3.2 ± 1.0	3.4 ± 0.9	0.055
17. Smartphone applications for university education will not be used by students unless they are involved during the development.	301	3.0 ± 1.1	3.2 ± 1.0	0.055
18. The more a university supports a smartphone application the more the students will use this technology.	299	3.9 ± 0.8	4.1 ± 0.8	**0.010**
19. Whether or not smartphone application will be used at a university depends on the trends and circumstances outside the universities.	300	3.1 ± 0.9	3.2 ± 0.9	0.472

**Table 3:** Students’ perception of the benefit and ease of use– group comparisons based on usage

Variable	Valid N	Five times or less used	More than five times used	P
		Mean ± SD	Mean ± SD	(*U*-Test)
20. How do you judge the benefit of the provided application for yourself? (*Scale from 1 = “small benefit“to 5 = “large benefit“*)	290	3.0 ± 1.2	3.8 ± 0.9	**<0.001**
21. The provided application is easy to use and not complicated. (*Likert scale from 1 = “strongly disagree” to 5 = “strongly agree“*)	288	3.7 ± 1.0	3.8 ± 1.0	0.682

### Multivariable analysis

A total of 16 variables were selected as potential predictors of frequent application usage (see Methods section) and entered into stepwise forward (LR) logistic regression. Included variables were gender, mobile device ownership, lecture attendance, exchange about certificate, personal benefit and all variables presented in Table 2 except for number 12 and 19. The resulting model is presented in Table 4.

**Table 4:** Multivariable binary logistic regression predicting more frequent application use (more than five times)

Variable	OR (95 % CI)	p
1. Gender female (vs. male)	2.09 (1.15 – 3.78)	**0.015**
22. How do you judge the benefit of the provided application for yourself? (*Scale from 1 = “small benefit“ to 5 = “large benefit“*)	1.99 (1.46 – 2.71)	**< 0.001**
7. I’m always belong to the first ones that use a new technology. (*Likert scale from 1 = “strongly disagree” to 5 = “strongly agree“*)	1.42 (1.07 –1.90)	**0.017**
16. Whether or not one uses a smartphone application essentially depends on previous related experiences. (*Likert scale from 1 = “strongly disagree” to 5 = “strongly agree“*)	1.43 (1.05 – 1.95)	**0.025**

The order of the variables corresponds to their contribution to explain the variance of the output variable. (N = 274 with valid values for all included variables, Pseudo-R^2^ = 0.245)

### Perceived adoption factors

**Fig. 1:**
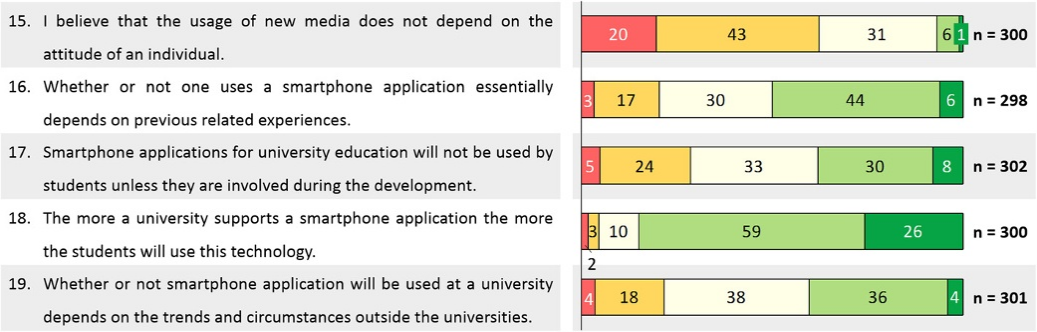
Frequency distributions for students’ agreement with selected attitudinal statements addressing app adoption. Values displayed as percentages. Colors reflect agreement on 5 point Likert scale from red (strongly disagree) to green (strongly agree)

As some of the variables (number 15 through 19) above also capture the student’s general opinion of what they consider relevant for adoption, it is also intriguing to mention those apart from the determinants of actual adoption. Figure 1 provides the frequency distributions for those variables to complement the mean and SD reported above. Regarding variables 16, 17 and 19 students from both groups on average choose a neutral agreement with the statement. However, when being asked about their agreement with the statement that the usage of new media does not depend on the attitude of an individual, 62.3 % (n = 187/300) disagreed. Furthermore, 85 % (n = 255/300) students believe that with higher university support of a smartphone application more students will use it.

## Discussion

### Main findings

Our study demonstrates that a majority of the surveyed medical students adopted a smartphone application for medical education. Furthermore it elicits that, based on multivariable analysis, female gender, a higher general interest in new technologies, a higher perceived benefit, as well as a higher perceived impact of previous experiences on smartphone application usage are independent predictors of frequent usage of an application specifically tailored for university education. In addition, eleven characteristics significantly distinguished frequent users from non-frequent users. With less than three percent of the students not having used the application at all, almost everyone has viewed it and then decided to either adopt or not adopt it.

### Discussion with findings from the literature

As similar studies are rare in the literature, we will discuss our findings on adoption with studies that researched adoption of smartphones in general and specifically by physicians as well as adoption of mobile-learning in general.

In our study, the female gender was the most important predictor of adopting our provided application. This is in contrast to a previous study examining predictors of physicians’ intention to use a smartphone [21]. Since our application was introduced in a course on general practice, this might be seen in the light of a higher interest of female students in pursuing a career as a general practitioner, as reported by previous studies [25, 26]. On the other hand, one could discuss that male and female students’ different learning styles, as described by current medical education studies, might have affected the usage behavior [27, 28]. Our finding of higher perceived personal benefit being a predictor of a more frequent application use is comparable to the results of other studies on mobile-learning and smartphones [19, 20]. A literature review summarized studies of the technology acceptance model in the broader healthcare domain. It was reported that 16 out of 16 studies found a significant relationship between perceived usefulness and behavioral intention. Two out of three of those studies also found the independent relationship between behavioral intention to use and actual use to be statistically significant [29]. Additionally consistent with previous studies is the relevance of personal innovativeness as an independent predictor [19].

The relevance of previous experiences was the fourth independent predictor revealed by the multivariate analysis. It is comparable to the innovation factor “compatibility”, which reportedly is significantly associated with physicians’ attitude towards using a smartphone [21, 22]. Contrary to other studies, the innovation factor of “observability” had no influence on the attitude towards a new technology even at the univariable level (mean of 3.5 ± 0.9 for frequent and 3.3 ± 1.1 for non-frequent, p = 0.164). In our study variable number 11 represents “observability”. We observed the same contrast for the variable 21 that reflects ease of use [20, 21]. We assume that this is due to the self-efficacy (indicated by variable 12) towards smartphones and applications among the surveyed students, which did not show a statistical difference between frequent users and non-frequent users (mean of 2.0 ± 1.0 for frequent and 2.1 ± 1.0 for non-frequent, p = 0.177). In other words, if users from both groups are similarly confident about their skills with the technology, they will also have a similar perception of the ease of use. The significant relationship between self-efficiency and perceived ease of use has been reported by Park and Chen [20].

The results on possible associations of application usage frequency with lecture attendance and exchange about the course certificate (Table 1) indicate, although only one being statistically significant, that general motivation for a course has an influence on adopting technologies provided in the courses context.

We found a different agreement (4.2 ± 0.8 vs. 3.8 ± 1.0, p < 0.001) between frequent and non-frequent users regarding the statement that medical smartphone applications are good for doctors. Furthermore, students from both groups agreed, although not as much as with the previous statement, significantly differently with the statement that medical smartphone applications are important in their working environment (2.8 ± 1.0 vs. 2.5 ± 1.0, p = 0.005). Thus, despite modern medical students considering smartphones as positive for doctors, this technology may not have gained full relevance in daily medical business yet. Barriers of smartphone implementation may contribute to this difference. For instance smartphones could be perceived as too costly or smartphone users could be perceived as unprofessional [30]. As proposed by Miller et al. this perception can be changed by actively educating the patient [31]. Furthermore, while students believed that application usage will grow with increasing university support on an aggregated level, those who used the application more frequently showed a significantly higher agreement than non-frequent users (4.1 ± 0.8 vs. 3.9 ± 0.8, p = 0.010). This highlights the importance of university support for a successful implementation strategy. It is worth mentioning that although our application has been designed as a mobile website that could also be used with a personal computer, mobile device ownership was significantly different between frequent and non-frequent users (83.5 vs. 72.2 %; p = 0.022). We interpret this as a sign of our application being more frequently used on smartphones or tablet computers.

### Strengths and limitations

We report an examination of factors associated with medical students adoption of a smartphone application specifically developed for their course by employing multivariable statistical methods. Our study provides sound findings in this highly innovative field. Furthermore, while most of the studies concerned with adoption of new technologies in the healthcare and mobile learning domain research independent predictors of behavioral intention to use, we have studied independent predictors of self-reported actual use [19, 21, 29]. Another strength of this study is the response rate of 99.3 % ensuring representativeness of the results. Additionally, the selection of questionnaire items has been based on previous innovation adoption research and therefore is comparable to existing studies.

A clear limitation of our study is the composition of the cohort comprising students from only one medical faculty in Germany. As our findings are related to a specific application prototype developed for medical education in general practice, the generalizability of our results may be limited. A further criticism could be that the outcome measure in this study, the more frequent use of the provided application, was based on students’ self-reports instead of user data collected by the application. However, we consider associated bias to be unlikely.

### Implications for education and future research

To ensure a wide adoption of a smartphone application for medical education, it must be ensured that the app provides the necessary medical information and functionality to ensure a high benefit to the students. Further research can build on our findings and should investigate possible determinants of adoption among cohorts of a different composition. Moreover, it would be interesting to see studies that research the adoption of different kinds of medical smartphone applications. In addition to only exploring the factors of adoption of one specific application it would be stimulating to see which functionalities of medical smartphone applications for education and practice promote higher adoption. Such a study for instance, could build an application with functionalities in areas as described by recent research on medical app usage in education and provide two cohorts with two different versions of an application [9, 32]. By employing this approach questions such as “Should a medical mobile-learning platform be combined with mockup-testing to increase adoption?” or “Should a medical application have collaboration or social functions linking different users to increase frequent usage?” could be answered. This would help universities and independent developers in guiding their efforts.

## Conclusions

As smartphone applications provide a wide spread spectrum of functionalities for medical education and the daily medical business, our study aimed to provide stakeholders of medical education with an understanding of factors related to the adoption of a mobile educational technology in the healthcare domain. Designers, developers and promoters have to be aware of the key characteristics of potential users to provide content and features that are congruent with user needs. By doing so, one can provide a useful and beneficial application leading to increased adoption among the target group. We believe that applications for mobile-learning related to university education, if done right, will more and more find their way into modern education.

## Additional files

Additional file 1:
**English translation of the questionnaire items relevant for the present analysis.**
Additional file 2:
**Screenshot of the user-interface of the provided smartphone application on a laptop and on a smartphone.**

## References

[1] Oehler RL, Smith K, Toney JF (2010). Infectious diseases resources for the iPhone. Clin Infect Dis..

[2] Gozalvez J (2008). First Google’s android phone launched [Mobile Radio]. IEEE Veh Technol Mag..

[3] Bort-Roig J, Gilson ND, Puig-Ribera A, Contreras RS, Trost SG (2014). Measuring and influencing physical activity with smartphone technology: a systematic review. Sports Med..

[4] Cho MJ, Sim JL, Hwang SY (2014). Development of smartphone educational application for patients with coronary artery disease. Healthc Inform Res..

[5] Markman TM, Sampognaro PJ, Mitchell SL, Weeks SR, Khalifian S, Dattilo JR (2013). Medical student appraisal: applications for bedside patient education. Appl Clin Inform..

[6] Nishiguchi S, Ito H, Yamada M, Yoshitomi H, Furu M, Ito T (2014). Self-assessment tool of disease activity of rheumatoid arthritis by using a smartphone application. Telemed J E Health..

[7] Balg F, Juteau M, Theoret C, Svotelis A, Grenier G (2014). Validity and Reliability of the iPhone to Measure Rib Hump in Scoliosis. J Pediatr Orthop..

[8] Handzel O, Ben-Ari O, Damian D, Priel MM, Cohen J, Himmelfarb M (2013). Smartphone-based hearing test as an aid in the initial evaluation of unilateral sudden sensorineural hearing loss. Audiol Neurootol..

[9] Sandholzer M, Rurik I, Deutsch T, Frese T (2014). Medical students’ expectations towards an implementation of a family medicine textbook as a comprehensive app in Germany. J Med Syst..

[10] Davies BS, Rafique J, Vincent TR, Fairclough J, Packer MH, Vincent R (2012). Mobile Medical Education (MoMEd) - how mobile information resources contribute to learning for undergraduate clinical students - a mixed methods study. BMC Med Educ..

[11] Boeder N: “Mediman” - the smartphone as a learning platform? GMS Z Med Ausbild 2013,30:Doc5. doi: 10.3205/zma000848, URN: urn:nbn:de:0183-zma0008484. 10.3205/zma000848 PMC3589687 23467633

[12] Waldmann U-M, Weckbecker K: Smartphone application of primary care guidelines used in education of medical students. GMS Z Med Ausbild. 2013;30(1):Doc6. doi: 10.3205/zma000849. 10.3205/zma000849 PMC3589688 23467657

[13] Zhang MW, Ho CS, Ho RC (2014). Methodology of development and students’ perceptions of a psychiatry educational smartphone application. Technol Heal care..

[14] Thorell M, Fridorff-Jens PK, Lassen P, Lange T, Kayser L (2015). Transforming students into digital academics: a challenge at both the individual and the institutional level. BMC Med Educ..

[15] Stanford School of Medicine: eStudent. http://med.stanford.edu/estudent (n.d.). Accessed 27 Mar 2014.

[16] University of California, Irvine: UCI’s iMedEd Initiative named a 2012–13 Apple Distinguished Program. http://news.uci.edu/press-releases/ucis-imeded-initiative-named-a-2012-13-apple-distinguished-program (2013). Accessed 27 Mar 2014.

[17] Trelease RB (2008). Diffusion of innovations: smartphones and wireless anatomy learning resources. Anat Sci Educ..

[18] Aldhaban F: Exploring the adoption of Smartphone technology: Literature review. In Technology Management for Emerging Technologies (PICMET), 2012 Proceedings of PICMET’12:; 2012:2758–2770

[19] Liu Y, Li H, Carlsson C (2010). Factors driving the adoption of m-learning: An empirical study. Comput Educ..

[20] Park Y, Chen JV (2007). Acceptance and adoption of the innovative use of smartphone. Ind Manag Data Syst Emerald..

[21] Putzer GJ, Park Y. Are physicians likely to adopt emerging mobile technologies? Attitudes and innovation factors affecting smartphone use in the Southeastern United States. Perspect Health Inf Manag. 2012;9:1b. PMC3329206 22737094

[22] Rogers EM (2003). Diffusion of Innovations.

[23] Kim SH (2008). Moderating effects of Job Relevance and Experience on mobile wireless technology acceptance: Adoption of a smartphone by individuals. Inf Manag..

[24] Sandholzer H, Frese T. Allgemeinmedizin: Familienmedizin. Aachen: Shaker Verlag; 2010

[25] Bennett KL, Phillips JP (2010). Finding, recruiting, and sustaining the future primary care physician workforce: a new theoretical model of specialty choice process. Acad Med.

[26] Roick C, Heider D, Günther OH, Kürstein B, Riedel-Heller SG, König HH (2012). Factors influencing the decision to establish a primary care practice: results from a postal survey of young physicians in Germany. Gesundheitswesen..

[27] Nuzhat A, Salem RO, Hamdan NA, Ashour N (2013). Gender differences in learning styles and academic performance of medical students in Saudi Arabia. Med Teach.

[28] Wehrwein EA, Lujan HL, DiCarlo SE (2007). Gender differences in learning style preferences among undergraduate physiology students. Adv Physiol Educ..

[29] Holden RJ, Karsh B-T (2010). The technology acceptance model: its past and its future in health care. J Biomed Inform..

[30] Robinson T, Cronin T, Ibrahim H, Jinks M, Molitor T, Newman J (2013). Smartphone use and acceptability among clinical medical students: a questionnaire-based study. J Med Syst..

[31] Miller KH, Ziegler C, Greenberg R, Patel PD, Carter MB (2012). Why Physicians Should Share PDA/Smartphone Findings With Their Patients: A Brief Report. J Health Commun.

[32] Payne KFB, Wharrad H, Watts K (2012). Smartphone and medical related App use among medical students and junior doctors in the United Kingdom (UK): a regional survey. BMC Med Inform Decis Mak..

