# Supplementary material for: Predictors of students’ self-reported adoption of a smartphone application for medical education in general practice
Source: BMC Med Educ. 2015 May 21;15:91. doi: 10.1186/s12909-015-0377-3 (PMC4460859; doi:10.1186/s12909-015-0377-3)
Supplement: Additional file 1: — English translation of the questionnaire items relevant for the present analysis. [file 12909_2015_377_MOESM1_ESM.docx]

1. Gender: 🞎 female 🞎 male

2. Age: years

3. I own the following technical devices or technologies:

🞎 Smartphone

🞎 Tablet computer

🞎 Laptop / Notebook

🞎 Desktop computer

🞎 Personal internet access

4. How often did you attend the lecture on general practice?

🞎 not at all 🞎 once 🞎 2 to 5 times 🞎 6 to 10 times 🞎 more than 10 times

5. How often have you used a medical textbook on general practice for the preparation and review of lectures?

🞎 not at all 🞎 once 🞎 2 to 5 times 🞎 6 to 10 times 🞎 more than 10 times

6. How often have you talked to your fellow students about obtaining the certificate of performance for the course on general practice?

🞎 not at all 🞎 once 🞎 2 to 5 times 🞎 6 to 10 times 🞎 more than 10 times

| To which extend do you agree with the following statements? | strongly disagree | disagree | neutral | agree | strongly agree |
| --- | --- | --- | --- | --- | --- |
| 1. I’m always belong to the first ones that use a new technology. | ➀ | ➁ | ➂ | ➃ | ➄ |
| 1. I do not enjoy the usage of smartphones and smartphone applications. | ➀ | ➁ | ➂ | ➃ | ➄ |
| 1. I will definitely use medical smartphone applications less in the future. | ➀ | ➁ | ➂ | ➃ | ➄ |
| 1. Using medical smartphone applications for the profession of a doctor is good. | ➀ | ➁ | ➂ | ➃ | ➄ |
| 1. Medical smartphone applications are used frequently among my fellow students. | ➀ | ➁ | ➂ | ➃ | ➄ |
| 1. I consider myself unconfident when handling smartphones and smartphone applications. | ➀ | ➁ | ➂ | ➃ | ➄ |
| 1. Having medical smartphone applications is important in my working environment. | ➀ | ➁ | ➂ | ➃ | ➄ |
| 1. Using medical smartphone applications does not fit personal way of working. | ➀ | ➁ | ➂ | ➃ | ➄ |
| 1. I believe that the usage of new media does not depend on the attitude of an individual. | ➀ | ➁ | ➂ | ➃ | ➄ |
| 1. Whether or not one uses a smartphone application essentially depends on previous related experiences. | ➀ | ➁ | ➂ | ➃ | ➄ |
| 1. Smartphone applications for university education will not be used by students unless they are involved during the development. | ➀ | ➁ | ➂ | ➃ | ➄ |
| 1. The more a university supports a smartphone application the more the students will use this technology. | ➀ | ➁ | ➂ | ➃ | ➄ |
| 1. Whether or not smartphone application will be used at a university depends on the trends and circumstances outside the universities. | ➀ | ➁ | ➂ | ➃ | ➄ |

20. How do you judge the benefit of “AllgemeinmedizinApp” for yourself?

| small benefit | ➀ ➁ ➂ ➃ ➄ | large benefit |
| --- | --- | --- |

21. „AllgemeinmedizinApp“ is easy to use and not complicated.

| Strongly disagree | ➀ ➁ ➂ ➃ ➄ | Strongly agree |
| --- | --- | --- |

22. How often have you used „AllgemeinmedizinApp“?

🞎 not at all 🞎 once 🞎 2 to 5 times 🞎 6 to 10 times 🞎 more than 10 times
