# Supplementary figures and images for: Predictors of students’ self-reported adoption of a smartphone application for medical education in general practice
Source: BMC Med Educ. 2015 May 21;15:91. doi: 10.1186/s12909-015-0377-3 (PMC4460859; doi:10.1186/s12909-015-0377-3)

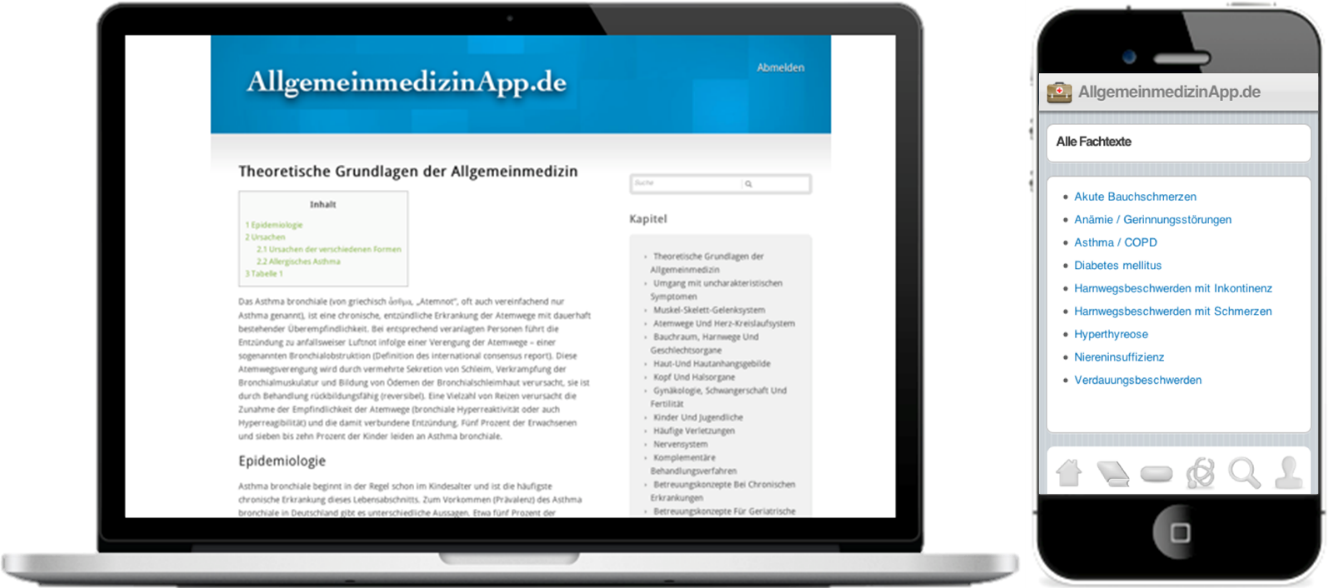

Supplement: Additional file 2: — Screenshot of the user-interface of the provided smartphone application on a laptop and on a smartphone. [file 12909_2015_377_MOESM2_ESM.png]
